# Diagnostic performance of Idylla MSI test in colorectal cancer biopsies

**DOI:** 10.1186/s13000-023-01328-6

**Published:** 2023-03-28

**Authors:** Iiris Ukkola, Pirjo Nummela, Mia Kero, Ari Ristimäki

**Affiliations:** 1 Department of Pathology, HUS Diagnostic Center, HUSLAB, Helsinki University Hospital, University of Helsinki, P.O. Box 400, FI-00029 HUS Helsinki, Finland; 2 Applied Tumor Genomics Research Program, Research Programs Unit, University of Helsinki, Helsinki University Hospital, Helsinki, Finland

**Keywords:** Biopsy, Colorectal cancer, Idylla, Immunohistochemistry, Microsatellite instability, Mismatch repair

## Abstract

Universal testing for microsatellite instability (MSI) is recommended in colorectal cancer (CRC) to screen for Lynch syndrome and to guide optimal treatment and follow-up of the patients. Especially in neoadjuvant setting, where immuno-oncological treatments have recently shown excellent responses, identification of MSI status at biopsy is a prerequisite. Idylla MSI test offers a rapid and automated test to assess MSI-status from formalin-fixed paraffin-embedded tumor tissue sections. In this study, we compared the performance of the Idylla MSI test to mismatch repair (MMR) protein immunohistochemistry (IHC) using 117 CRC biopsies with previously known deficient MMR status. The concordance between Idylla and IHC was 99.0% (95/96) for biopsies with the recommended ≥ 20% tumor cell content. Further, 85.7% (18/21) of suboptimal CRC biopsy specimens (tumor cell content 5–15%) were diagnosed as MSI. Overall, we identified four discrepant cases of which three had tumor cell content less than 20%, explaining the discordant result. Our study shows that the Idylla MSI test offers a competent tool for MSI screening in CRC biopsy specimens.

**Supplementary Information:**

The online version contains supplementary material available at 10.1186/s13000-023-01328-6.

## Introduction

Microsatellites are small elements of repeated DNA, which are prone to form mismatches during DNA replication. DNA mismatch repair (MMR) system encoded by the MMR genes (*MLH1, MSH2, MSH6*, and *PMS2*) normally functions to correct these replication errors. Deficient MMR (dMMR) system leads to microsatellite instability (MSI) and hypermutation phenomenon causing increased cancer susceptibility [1, 2]. The MMR system can be compromised by epigenetic mechanism, usually by acquired *MLH1* promoter hypermethylation, or by genetic inactivation characteristic to Lynch syndrome (LS). MSI has been identified in approximately 15% of colorectal cancer (CRC) cases, of which approximately 80% are sporadic [3].

Universal testing for dMMR/MSI has been recommended in CRC to screen for LS and to guide optimal follow-up and treatment for the patients [4–6]. It can be critical to analyze dMMR/MSI status from pre-treatment rectal cancer biopsy specimens, considering the loss of tumor burden due to successful neoadjuvant treatment and possible post-neoadjuvant absence of MSH6 protein expression [1, 4, 6]. Since immuno-oncological treatments have shown notable effectiveness in treating advanced dMMR/MSI CRC patients [4, 6], and recently also in the neoadjuvant setting with excellent responses [7], identification of dMMR/MSI status at initial diagnosis using biopsy samples has been proposed to be necessary diagnostic procedure [8].

The two most general methods to detect dMMR/MSI in CRC are MMR IHC and polymerase chain reaction (PCR)-based microsatellite tests, of which IHC is widely used as gold standard in pathology laboratories [4]. Both methods can also be used to complement each other in diagnostically challenging cases and to confirm the MSI status before starting immuno-oncological treatments [4, 6]. IHC is the most affordable method but requires trained personnel and laborious hands-on time and is also prone to pitfalls in pre-analytical and analytical phases. It can, however, be used on biopsy samples with low tumor cell content. PCR-based microsatellite tests, on the other hand, may have sensitivity issues with low tumor cell percentage or low DNA purity/yield.

Idylla MSI test is a novel real-time PCR based assay which analyzes a set of seven microsatellite marker specific probes (*ACVR2A*, *BTBD7*, *DIDO1*, *MRE11*, *RYR3*, *SEC31A*, and *SULF2*) using fluorescent-labeled molecular beacons and melting curve analysis. Idylla test is a rapid way to assess MSI/MSS status from formalin-fixed paraffin-embedded (FFPE) tissue sections, comprising automatic DNA extraction, PCR amplification, software interpretation and reporting. The specificity and sensitivity of Idylla MSI test to detect MSI status has been shown to be as high as 98–100% and 94–100% respectively evaluated mainly from surgical CRC specimens and with tumor cell content over 20% [9–11].

Since the diagnostic performance of Idylla MSI test has previously been shown to be optimal in CRC surgical resection specimens, especially giving no false positives [9, 11], we here wanted to scrutinize the usability of Idylla MSI test in CRC biopsies. For that, we compared the performance of the Idylla MSI test to the gold standard MMR IHC in 117 CRC biopsy samples with known dMMR status.

## Materials and methods

### Sample selection

We analyzed 117 colonoscopy biopsies with known dMMR status from CRC patients, who had biopsies taken in The Hospital District of Helsinki and Uusimaa between October 2019 and December 2021. The patients had not undergone any neoadjuvant treatment prior the colonoscopy and the tumors were routinely screened for MMR proteins MLH1, MSH2, MSH6, and PMS2 using IHC. The set of CRC biopsies included 84 adenocarcinomas not otherwise specified (NOS), 13 partim mucinosum adenocarcinomas (less than 50% mucinous component), two mucinous adenocarcinomas (50% or more mucinous component), five signet ring cell carcinomas (50% or more signet ring cells), four adenocarcinomas with signet ring differentiation (less than 50% signet ring cells), eight adenomas with minimal invasive component and one large adenoma with high-grade dysplasia. The study was approved by the Ethics Committee of the HUH.

### Immunohistochemistry

All 117 CRC biopsies had undergone diagnostic IHC to detect the loss of MMR protein expression as a gold standard and the method was performed as described previously [11]. The absence of one or more MMR protein expression with positive external and internal controls was considered as dMMR. The dMMR status of the biopsies was re-analyzed in a blinded manner by AR and IU, and all cases were confirmed to be dMMR.

### Idylla MSI test

We analyzed the 117 dMMR CRC biopsies with automated Idylla MSI test, according to the manufacturer’s protocol. The recommended tumor cell percentage for Idylla MSI test is ≥ 20% for CRC samples and the required total tissue area is 25–300 mm^2^ using 10 μm thick sections. For the analysis, one to four 10 μm sections were cut from the FFPE biopsy tissue blocks with a Leica SM2000R microtome (Leica Microsystems GmbH, Wetzlar, Germany). The tumor cell percentage was estimated from the biopsy HE slides by two independent observers (AR and IU) both before and after cutting the tissue sections for Idylla. Macrodissection was performed only for two biopsy samples where the non-neoplastic colon areas were clearly separate from the cancerous areas. The tumor cell percentages varied between 5 and 80% of which 21 samples had < 20% tumor content. Detection of two or more mutant microsatellite markers (*ACVR2A*, *BTBD7*, *DIDO1*, *MRE11*, *RYR3*, *SEC31A*, and/or *SULF2*) using the Idylla MSI test is classified as MSI, whereas less than two mutant markers lead to MSS result. MSI score cutoff value 0.5 is used to judge the marker as mutated.

## Results

Our dMMR CRC biopsy set (n = 117) consisted of patients with median age of 76 years (range from 27 to 99) with slight female predominance (56.4%). Tumors were mainly low-grade (72.6%) and localized to the right colon (69.2%), the proportion of rectum tumors being 10.3%. Majority of the cases represented dMLH1 (85.5%) with minority of other dMMR subclasses (dMSH2 9.4%, dMSH6 2.6% and dPMS2 2.6%).

We analyzed the 117 CRC biopsies by Idylla MSI test and the concordance between Idylla and MMR IHC was 96.6% (113/117) among all the cases (5–80% tumor cell percentage) and 99.0% (95/96) among the cases with the recommended ≥ 20% tumor cell content. Of the four discrepant cases, three contained tumor cell percentage from 5 to 15% and the remaining fourth case (C40) had tumor cell percentage 40–50%, estimated from pre- and/or post-Idylla HE slides (Table 1). There were no invalid results, but one cassette failure occurred in the study set and a re-analysis was needed for that sample.

**Table 1:** Characteristics of the four discrepant CRC biopsy cases between MMR IHC and Idylla MSI test

Case	Tumor site	Tumor type	Tumor cell% pre/post Idylla	Tumor area mm^2^	MMR IHC	Idylla 1st analysis	Idylla 2nd analysis*
C19	Left	NOS	15/10	25	dMLH1/PMS2	MSS	MSS
C38	Left	partim mucinosum	30/10	25	dMLH1/PMS2	MSS	MSS
C40	Left	signet ring cell	50/40	25	dMSH2/dMSH6	MSS	MSI
C70	Right	signet ring cell	5/5	25	dMLH1/PMS2	MSS	MSS

*2nd analysis was performed with an increased number of formalin-fixed paraffin-embedded sections.

Colorectal cancer (CRC), Mismatch repair (MMR), Microsatellite instable (MSI), Microsatellite stable (MSS), Not otherwise specified (NOS).

**Fig. 1:**
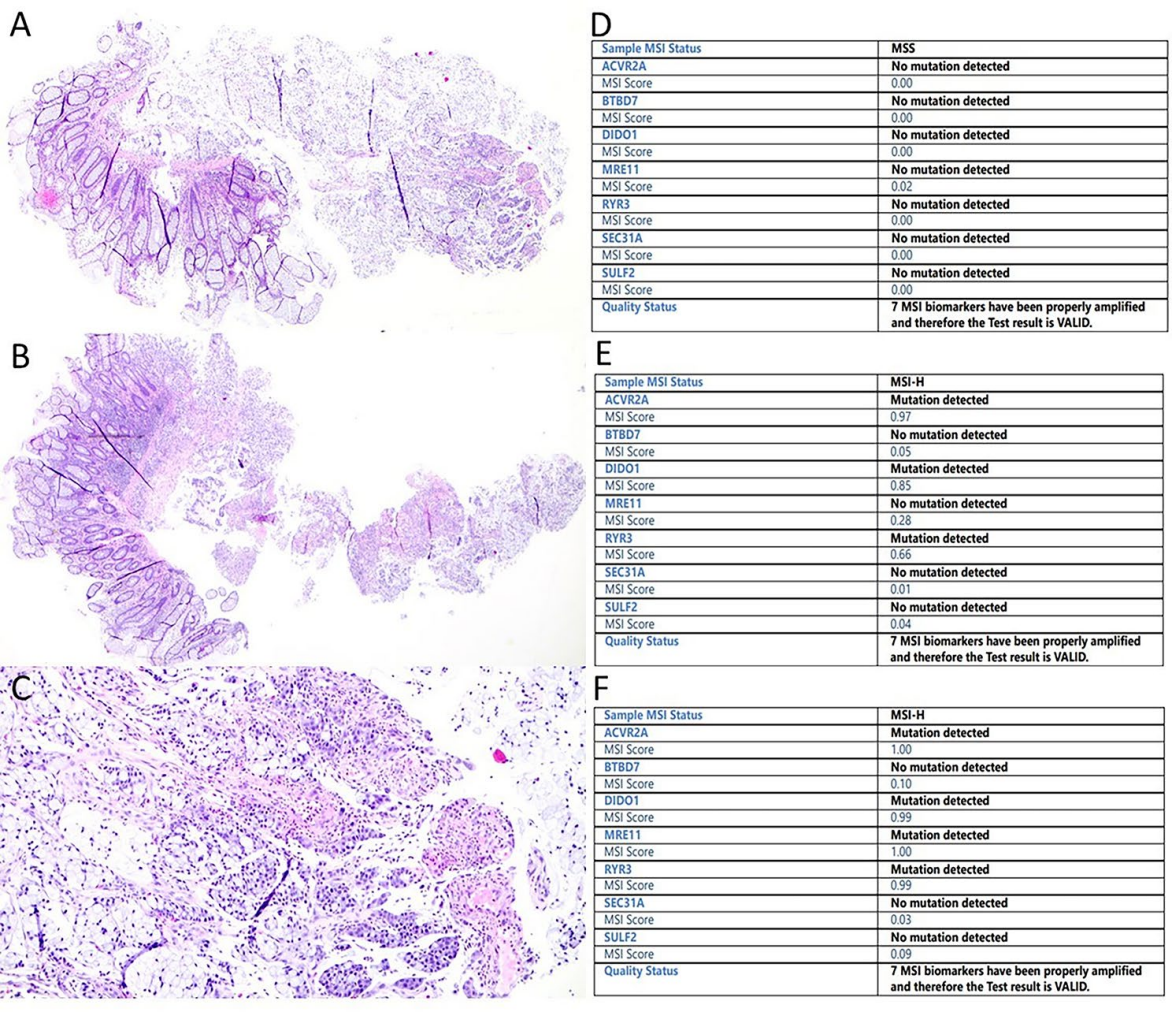
Histology and Idylla MSI test reports of the signet ring cell carcinoma case (C40). (A, B) Hematoxylin-eosin staining of the two biopsies in the analyzed tissue block (original magnification x20) (C) Cancer cell area of the case C40 (original magnification x100). (D-F) Idylla MSI test report of the first analysis (two flakes, D), of the second analysis (three flakes, E), and of the surgical resection specimen (one flake, F)

**Fig. 2:**
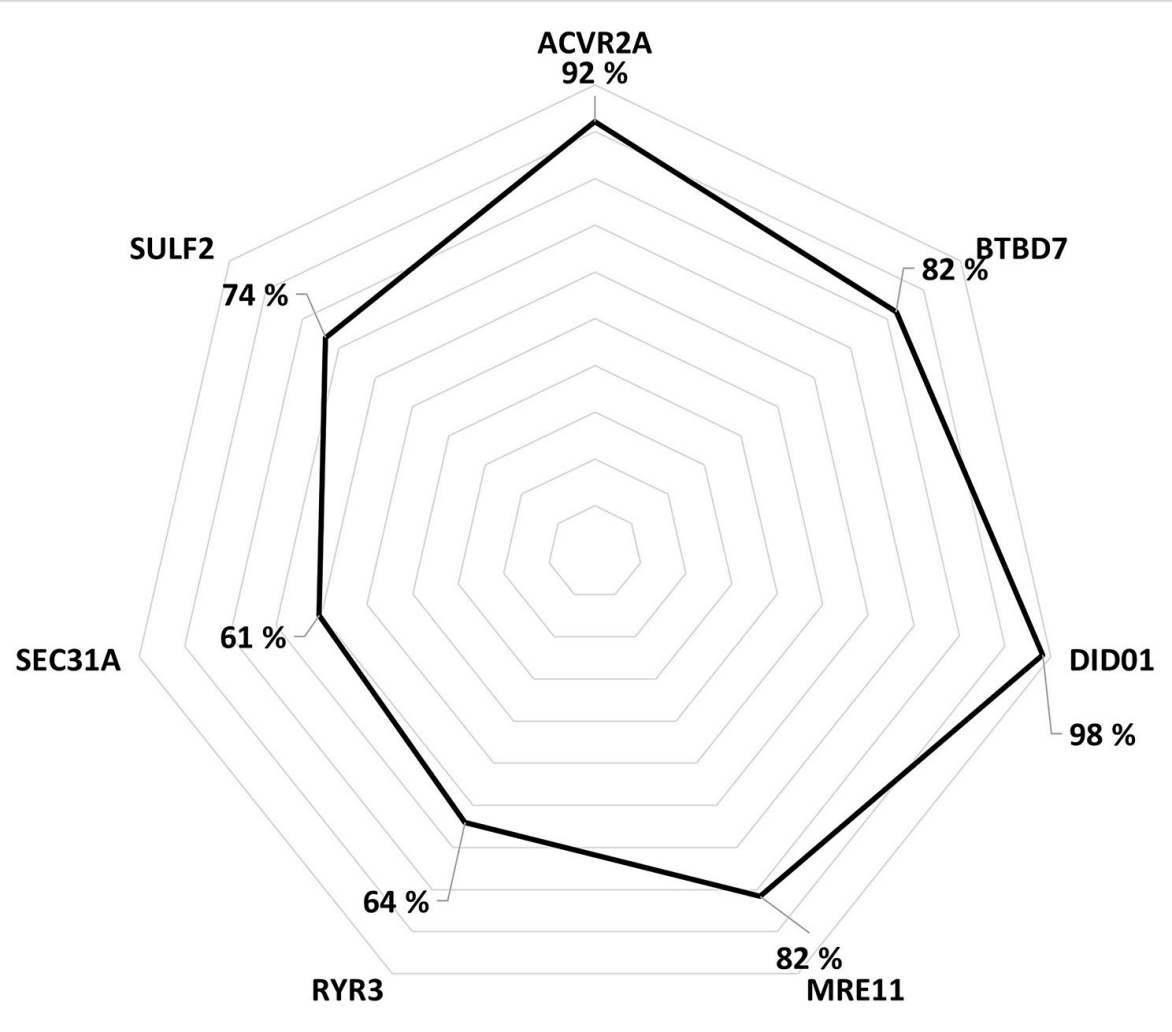
Mutated biomarker spectrum in colorectal cancer biopsies detected MSI (n = 114) by Idylla MSI test

We next re-analysed all the four discrepant biopsies with Idylla using increased number of sections (from two sections to three to four sections) and the three suboptimal cases remained discrepant (MSS), whereas the result for the case C40 changed to MSI (Fig. 1; Table 1). All discrepant cases had 0/7 microsatellite markers mutated (Supplementary Figure S1). We further analyzed the surgical resection CRC samples of the discrepant cases with tumor cell content of > 20% by Idylla and all the results were confirmed to be MSI. In cases classified MSI by Idylla, the most commonly mutated markers were *DIDO1* and *ACVR2A* with frequencies of 98% and 92%, respectively (Fig. 2).

## Discussion

We here evaluated the diagnostic performance of Idylla MSI test to detect MSI in CRC biopsy specimens as compared to MMR IHC. For that we analyzed 117 dMMR CRC biopsies. The concordance between Idylla and IHC was 99.0% (95/96) with the cases having recommended ≥ 20% tumor cell content. However, it was 96.6% (113/117) in the whole set including also the 21 less optimal cases with < 20% tumor cell content. The weakness of this study was that we did not include any proficient MMR CRC biopsies to the study and therefore could not evaluate the specificity of the Idylla MSI test in CRC biopsy material. The study focused on evaluation of the performance of Idylla test only in IHC dMMR CRC biopsies, because the optimal specificity (98–100%) of the Idylla MSI test has been repeatedly confirmed in CRC and the sensitivity (94–100%) with false negative results has been more often compromised with Idylla when compared to IHC [9–11].

We identified four discrepant cases from which 3/4 had pre- and/or post Idylla tumor cell content less than 20%, explaining the repeated MSS result in CRC biopsy specimens. One initially discrepant case (C40) with an adequate tumor cell percentage was detected MSI only after re-analysis with increased number of sections. This case represented signet ring cell carcinoma from which the tumor cell content can be challenging to evaluate (Fig. 1), and this histological type of the tumor may require increased number of tissue flakes for successful analysis. Defective molecular testing has also previously been reported in cancer samples with mucinous component leading to poor DNA quality [12].

Our findings of the excellent diagnostic performance of the Idylla MSI test in CRC biopsy material is in line with previous studies where the MSI/MSS status has been evaluated mainly from surgical CRC specimens. The concordance across Idylla and IHC has been previously reported to be 96–100% with 100% specificity in CRC with tumor cell content ≥ 20% [9–11, 13]. In our MSI CRC biopsy cases detected by the Idylla, the least mutated biomarker (61%) was *SEC31A* concordant with previous studies [10, 11]. Differing from the previous reports the most often mutated marker was *DIDO1* (98%) and *ACVR2A* (92%) was only the second most mutated. Good accuracy of Idylla MSI test has also been confirmed in biopsy material of gastric adenocarcinoma by Farmkiss et al. scoring 96% concordance and 100% specificity between Idylla and IHC (n = 50) [14]. To our knowledge this is the first study to evaluate the diagnostic performance of the Idylla MSI test in CRC biopsy material.

Our study confirms that the Idylla MSI test is an accurate diagnostic method to identify MSI status also in CRC biopsy samples with ≥ 20% tumor cell content. Biopsies of signet ring cell carcinomas may need increased number of tissue flakes for the analysis even with instructed tumor cell percentage. We can conclude that the Idylla MSI test offers a competent tool for MSI screening in CRC biopsies.

## Electronic supplementary material

Below is the link to the electronic supplementary material.

Supplementary Material 1

## Abbreviations

CRC: colorectal cancer
dMMR: deficient MMR
FFPE: formalin-fixed paraffin-embedded
HUH: Helsinki University Hospital
IHC: immunohistochemistry
LS: Lynch syndrome
MMR: mismatch repair
MSI: microsatellite instability
MSS: microsatellite stable
pMMR: proficient MMR
