# Supplementary material for: Diagnostic performance of Idylla MSI test in colorectal cancer biopsies
Source: Diagn Pathol. 2023 Mar 28;18:39. doi: 10.1186/s13000-023-01328-6 (PMC10053848; doi:10.1186/s13000-023-01328-6)
Supplement: Supplementary file 1 — Supplementary Material 1 [file 13000_2023_1328_MOESM1_ESM.pdf]

Supplementary Information

for

Diagnostic performance of Idylla MSI test in colorectal cancer biopsies

Iiris Ukkola[1,2], Pirjo Nummela[2], Mia Kero[1], Ari Ristimäki[1,2*]

[1]Department of Pathology, HUSLAB, HUS Diagnostic Center, Helsinki University Hospital and University of Helsinki, Helsinki, Finland

[2]Applied Tumor Genomics Research Program, Research Programs Unit, University of Helsinki and Helsinki University Hospital, Helsinki, Finland

A

Sample MSI Status	MSS
ACVR2A	No mutation detected
MSI Score	0.01
BTBD7	No mutation detected
MSI Score	0.02
DIDO1	No mutation detected
MSI Score	0.00
MRE11	No mutation detected
MSI Score	0.07
RYR3	No mutation detected
MSI Score	0.00
SEC31A	No mutation detected
MSI Score	0.00
SULF2	No mutation detected
MSI Score	0.00
Quality Status	7 MSI biomarkers have been properly amplified and therefore the Test result is VALID.

B

Sample MSI Status	MSS
ACVR2A	No mutation detected
MSI Score	0.00
BTBD7	No mutation detected
MSI Score	0.00
DIDO1	No mutation detected
MSI Score	0.00
MRE11	No mutation detected
MSI Score	0.01
RYR3	No mutation detected
MSI Score	0.00
SEC31A	No mutation detected
MSI Score	0.00
SULF2	No mutation detected
MSI Score	0.00
Quality Status	7 MSI biomarkers have been properly amplified and therefore the Test result is VALID.

C

Sample MSI Status	MSI-H
ACVR2A	Mutation detected
MSI Score	1.00
BTBD7	Mutation detected
MSI Score	1.00
DIDO1	Mutation detected
MSI Score	0.96
MRE11	Mutation detected
MSI Score	0.63
RYR3	Mutation detected
MSI Score	0.98
SEC31A	No mutation detected
MSI Score	0.01
SULF2	Mutation detected
MSI Score	0.97
Quality Status	7 MSI biomarkers have been properly amplified and therefore the Test result is VALID.

D

**Sample MSI Status**	**MSS**
**ACVR2A**	**No mutation detected**
MSI Score	0.01
**BTBD7**	**No mutation detected**
MSI Score	0.00
**DIDO1**	**No mutation detected**
MSI Score	0.01
**MRE11**	**No mutation detected**
MSI Score	0.03
**RYR3**	**No mutation detected**
MSI Score	0.00
**SEC31A**	**No mutation detected**
MSI Score	0.00
**SULF2**	**No mutation detected**
MSI Score	0.00
**Quality Status**	**7 MSI biomarkers have been properly amplified and therefore the Test result is VALID.**

E

**Sample MSI Status**	**MSS**
**ACVR2A**	**No mutation detected**
MSI Score	0.00
**BTBD7**	**No mutation detected**
MSI Score	0.00
**DIDO1**	**No mutation detected**
MSI Score	0.00
**MRE11**	**No mutation detected**
MSI Score	0.01
**RYR3**	**No mutation detected**
MSI Score	0.00
**SEC31A**	**No mutation detected**
MSI Score	0.00
**SULF2**	**No mutation detected**
MSI Score	0.00
**Quality Status**	**7 MSI biomarkers have been properly amplified and therefore the Test result is VALID.**

F

**Sample MSI Status**	**MSI-H**
**ACVR2A**	**Mutation detected**
MSI Score	1.00
**BTBD7**	**Mutation detected**
MSI Score	1.00
**DIDO1**	**Mutation detected**
MSI Score	0.97
**MRE11**	**Mutation detected**
MSI Score	1.00
**RYR3**	**Mutation detected**
MSI Score	0.99
**SEC31A**	**Mutation detected**
MSI Score	1.00
**SULF2**	**Mutation detected**
MSI Score	1.00
**Quality Status**	**7 MSI biomarkers have been properly amplified and therefore the Test result is VALID.**

G

Sample MSI Status	MSS
ACVR2A	No mutation detected
MSI Score	0.01
BTBD7	No mutation detected
MSI Score	0.00
DIDO1	No mutation detected
MSI Score	0.01
MRE11	No mutation detected
MSI Score	0.02
RYR3	No mutation detected
MSI Score	0.00
SEC31A	No mutation detected
MSI Score	0.00
SULF2	No mutation detected
MSI Score	0.00
Quality Status	7 MSI biomarkers have been properly amplified and therefore the Test result is VALID.

H

Sample MSI Status	MSS
ACVR2A	Mutation detected
MSI Score	0.99
BTBD7	No mutation detected
MSI Score	0.21
DIDO1	No mutation detected
MSI Score	0.35
MRE11	No mutation detected
MSI Score	0.16
RYR3	No mutation detected
MSI Score	0.02
SEC31A	No mutation detected
MSI Score	0.00
SULF2	No mutation detected
MSI Score	0.04
Quality Status	7 MSI biomarkers have been properly amplified and therefore the Test result is VALID.

I

Sample MSI Status	MSI-H
ACVR2A	Mutation detected
MSI Score	0.90
BTBD7	No mutation detected
MSI Score	0.15
DIDO1	No mutation detected
MSI Score	0.19
MRE11	Mutation detected
MSI Score	0.86
RYR3	No mutation detected
MSI Score	0.04
SEC31A	No mutation detected
MSI Score	0.00
SULF2	No mutation detected
MSI Score	0.14
Quality Status	7 MSI biomarkers have been properly amplified and therefore the Test result is VALID.

Supplementary Figure S1. Idylla MSI test reports of the discrepant biopsies of the cases C19, C38, and C70. (A-C) Case 19 Idylla MSI test report of the first analysis (two flakes, A), of the second analysis (three flakes, B), and of the surgical resection sample (one flake, C). (D-F) Case 38 Idylla MSI test report of the first analysis (two flakes, D), of the second analysis (four flakes, E), and of the surgical resection sample (one flake, F). (G-I) Case 70 Idylla MSI test report of the first analysis (two flakes, G), of the second analysis (four flakes, H), and of the surgical resection sample (one flake, I).
